# Clinical utility index for root canal sealers

**DOI:** 10.1186/s12903-024-05047-2

**Published:** 2024-10-21

**Authors:** Manoj Chandak, Aditya Patel, Satyawansingh Patel, Paridhi Agrawal, Rakhi Chandak, Anuja Ikhar

**Affiliations:** 1 Department of Conservative Dentistry and Endodontics, Sharad Pawar Dental College and Hospital, Datta Meghe Institute of Higher Education and Research, Wardha, Maharashtra 442107 India; 2 Department of Public Health, Jawaharlal Nehru Medical College, Datta Meghe Institute of Higher Education and Research, Wardha, Maharashtra 442107 India; 3 Department of Oral Medicine and Radiology, Sharad Pawar Dental College and Hospital, Datta Meghe Institute of Higher Education and Research, Wardha, Maharashtra 442107 India

**Keywords:** Antimicrobial efficacy, Clinical utility index, Endodontics, Flow rate, Root canal sealer, Sealing ability, Solubility

## Abstract

**Background:**

Effective endodontic treatment requires the use of a root canal sealer with optimal properties to ensure a hermetic seal, prevent reinfection, and promote healing. Despite the availability of various sealers, a standardized evaluation system still needs to be improved.

**Objectives:**

To develop a Clinical Utility Index (CUI) that systematically evaluates and ranks root canal sealers based on their sealing ability, antimicrobial efficacy, flow rate, and solubility.

**Methods:**

The CUI was developed through a structured process involving expert identification, panel discussions, and the establishment of scoring criteria. Five sealers were evaluated (Sealers A, B, C, D, and E). Mean values for the core properties were calculated, and sealers were ranked accordingly. The total CUI for each sealer was computed based on the assigned scores for each property.

**Results:**

Sealer B achieved the highest CUI at 95%, demonstrating superior performance across all core properties. Sealer C followed with a CUI of 80%, while Sealer A ranked third with 60%. Sealers D and E showed the lowest performance, with CUIs of 30% and 35%, respectively, highlighting deficiencies in multiple properties.

**Discussion:**

The CUI provides a comprehensive evaluation framework for root canal sealers, facilitating informed decision-making by practitioners. Sealer B's high CUI underscores the importance of balancing sealing ability, antimicrobial effect, flow rate, and solubility. The results align with existing literature emphasizing the critical role of these properties in endodontic success.

**Conclusion:**

The CUI offers a robust and balanced method for evaluating root canal sealers, aiding in selecting the most suitable sealer based on empirical data. Future research should refine the index and validate its applicability in diverse clinical scenarios to enhance endodontic treatment outcomes.

## Introduction

Endodontic therapy, commonly known as root canal treatment, is a critical procedure designed to treat diseased dental pulp, aiming to eliminate infection and prevent further microbial invasion. This treatment involves both chemical and mechanical preparation of the root canal system to allow for the placement of a biocompatible material that achieves a three-dimensional hermetic seal. The essential steps in root canal preparation include access cavity preparation, establishing the glide path for instrumentation, thorough cleaning and irrigation of the root canals, and finally, three-dimensional obturation of the canal space [1].

One of the fundamental components of root canal obturation is the endodontic sealer, which works alongside the core material, typically "Gutta Percha." The sealer fills spaces between the core material and the canal walls, as well as any accessory canals, ensuring a fluid-tight seal [2]. It also facilitates lubrication during obturation and forms bonds between the filling material and dentinal structures, which are essential for preventing microleakage and promoting the healing of periapical lesions [3]. The ideal sealer should be biocompatible, dimensionally stable, and provide a strong seal to prevent microleakage [4].

Despite the availability of various root canal sealers, failures in endodontic treatment can still occur. These failures are primarily categorized as physical or biological. Physical failures often result from improper flow, high solubility, or inadequate sealing ability of the sealer. Biological failures typically stem from reinfections caused by persistent pathogens such as *Enterococcus faecalis* (24–77%) and *Candida albicans* (0.5–1.5%) [5, 6].

Currently, there is no standardized index to rank the clinical utility of the numerous available sealers. This lack of a comprehensive evaluation system complicates the selection process for practitioners, as they must rely on individual property assessments rather than an integrated approach [7]. Essential properties such as sealing ability, antimicrobial effect, flow rate, and solubility are critical for the success of a sealer. These four core properties must be balanced to ensure the effectiveness and longevity of root canal treatments [8, 9]. To address this gap, our study aims to develop a Clinical Utility Index (CUI) that evaluates these core properties, thereby assisting practitioners in selecting the most suitable sealers for clinical use.

## Material and method

### Development of clinical utility index

A Clinical Utility Index was devised to assess and select a sealer based on the four core properties. The primary objective behind formulating this index was to assign appropriate weightage to the four identified core properties of root canal sealers. This index would facilitate a systematic and quantitative evaluation, enabling practitioners to make informed decisions regarding the clinical suitability of different sealers based on their specific attributes.

The index development process unfolded through several meticulous steps-

- Step 1- Identification of experts
- A group of qualified individuals was carefully chosen for their expertise in the field. This step aimed to assemble a panel of knowledgeable professionals capable of contributing valuable insights to the subsequent phases of the index development, resulting in the appointment of the following distinguished experts. Each expert brought a unique set of skills and experiences to the table, ensuring a comprehensive and well-rounded perspective during the development of the Clinical Utility Index.

- Step 2- Panel discussion
- A substantive panel discussion ensued, leading to the unanimous decision to allocate equal weightage to all four core properties identified for root canal sealers. This consensus meant that the Clinical Utility Index would assess the performance of each sealer evenly across antimicrobial efficacy, flow characteristics, solubility resistance, and sealing ability. Consequently, the overall clinical utility of a sealer would be contingent on its holistic performance, with each core property contributing equally to the evaluation process. This strategic approach aimed at fostering a balanced and comprehensive assessment of root canal sealers within the Clinical Utility Index framework.

- Step 3: Scoring criteria for clinical utility index
- Mean values for each property were calculated and recorded to compare different sealers (Sealer A, Sealer B, Sealer C, Sealer D, and Sealer E). Each sealer was ranked based on its mean value for each property, and scores were assigned accordingly. The total Clinical Utility Index (in %) was computed using a formula considering the scores across all four core properties.
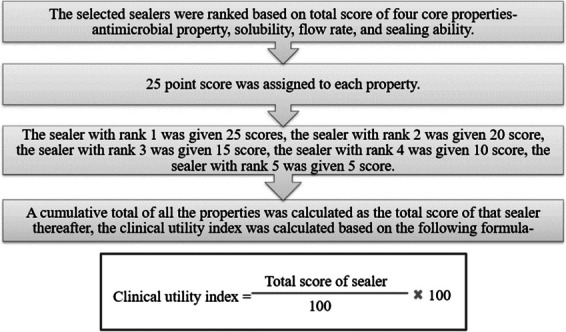
- Like, for example, in comparing five different sealers (Sealer 1, Sealer 2, Sealer 3, Sealer 4, and Sealer 5), mean values for each of the four properties are calculated and recorded in the table. Subsequently, each sealer is individually ranked based on its mean value for each property, and scores are assigned accordingly. Following this ranking process, the total Clinical Utility Index (in %) is computed using the formula mentioned above that considers the scores across all four core properties.

**Table:** 

	**Antimicrobial efficacy**		**Solubility**		**Flow rate**		**Sealing ability**		**Clinical utility index**
	Rank	**Score**	Rank	**Score**	Rank	**Score**	Rank	**Score**	
**Sealer A**	3	**15**	4	**10**	3	**15**	2	**20**	**60%**
**Sealer B**	1	**25**	1	**25**	2	**20**	1	**25**	**95%**
**Sealer C**	2	**20**	2	**20**	1	**25**	3	**15**	**80%**
**Sealer D**	5	**5**	3	**15**	5	**5**	5	**5**	**30%**
**Sealer E**	4	**10**	5	**5**	4	**10**	4	**10**	**35%**

## Results

The Clinical Utility Index provided a comprehensive and quantitative evaluation of the five different root canal sealers. Sealer B achieved the highest Clinical Utility Index at 95%, demonstrating superior performance across all four core properties. This sealer exhibited excellent antimicrobial efficacy, minimal solubility, optimal flow rate, and exceptional sealing ability.

Sealer C followed with a Clinical Utility Index of 80%, showing strong performance in most areas but slightly lower scores in sealing ability compared to Sealer B. Sealer A ranked third with a 60% Clinical Utility Index, performing moderately well across all properties but lacking in solubility resistance.

Sealer D and Sealer E showed the lowest performance, with Clinical Utility Indices of 30% and 35%, respectively. These sealers had significant weaknesses in multiple properties, particularly in flow rate and sealing ability, highlighting the need for improvements in these areas.

## Discussion

The development of a Clinical Utility Index (CUI) for root canal sealers marks a significant advancement in the field of endodontics, providing a comprehensive and standardized method for evaluating the effectiveness of various sealers. The Clinical Utility Index (CUI) developed in this study is primarily based on a comprehensive literature search and the opinions of experts in the field. While we reviewed existing studies to identify key parameters that influence the performance of root canal sealers, the final formulation of the index was guided by the consensus of the expert panel. Their insights were instrumental in prioritizing the most relevant properties for evaluation, ensuring that the CUI reflects both empirical evidence and expert judgment. This approach enhances the relevance and applicability of the index in clinical settings.

The expert team assembled for the development of the Clinical Utility Index consisted of distinguished professionals, each contributing unique expertise to ensure a comprehensive evaluation of root canal sealers. The panel included endodontists with extensive clinical experience, who provided insights into the practical applications and challenges associated with different sealers in everyday practice. Researchers specializing in dental materials contributed valuable knowledge regarding the physical and chemical properties of sealers, ensuring that the index reflects evidence-based findings. Additionally, biostatisticians were involved in establishing the scoring criteria, ensuring that the data analysis was robust and scientifically sound. The collaborative panel discussions led to a unanimous decision to allocate equal weightage to the four core properties—antimicrobial efficacy, flow characteristics, solubility resistance, and sealing ability. This consensus was pivotal in shaping the Clinical Utility Index, allowing for a balanced assessment that would facilitate informed decision-making by practitioners.

The mean values for each core property can be derived based on a standardized protocol developed for evaluating individual properties of root canal sealers. This approach ensures that while comparing the sealers, they are evaluated using the same methodology, allowing for consistent and fair comparisons across all properties.

The CUI is particularly valuable because it considers multiple critical properties—sealing ability, antimicrobial effect, flow rate, and solubility—all of which play a vital role in the overall success of root canal treatment.

### Sealing ability

The ability of a sealer to create a fluid-tight seal is critical in preventing microleakage, which can lead to treatment failure. Sealers with superior sealing ability ensure that the root canal system remains isolated from the oral environment, thereby preventing reinfection and promoting periapical healing. Our findings correlate with previous studies, where resin-based sealers have consistently demonstrated superior sealing properties [10, 11].

### Antimicrobial effect

Persistent infection is a major cause of endodontic failure, often due to the presence of bacteria such as *Enterococcus faecalis* and fungi like *Candida albicans*. Sealers with strong antimicrobial properties can significantly reduce the canal's microbial load, enhancing treatment success. In our study, we observed that antimicrobial activity was particularly crucial in cases where mechanical debridement alone was insufficient to eliminate pathogens [9, 12].

### Flow rate

The flow characteristics of a sealer determine its ability to penetrate into irregularities and accessory canals. A sealer with optimal flow can ensure thorough filling and adaptation to the canal walls, which is essential for a complete seal. Poor flow properties can result in voids and gaps, compromising the integrity of the obturation. Our results show that bio-ceramic sealers, in line with previous findings, exhibited excellent flow properties, contributing to effective sealing [13, 14].

### Solubility

Low solubility is essential for maintaining the integrity of the sealer over time. A sealer that dissolves or degrades can create spaces for microbial infiltration, leading to reinfection and treatment failure. In line with prior research, sealers with low solubility rates ensure greater durability and long-term effectiveness in root canal therapy [15, 16].

The exclusion of biocompatibility and periapical healing potential from this study's evaluation was based on recommendations from an expert panel and a thorough literature review. While these parameters are indeed important in assessing root canal sealers, the panel advised prioritizing parameters that have a more immediate impact on clinical effectiveness. Biocompatibility assessments typically require extensive biological testing and long-term evaluations, which were beyond the scope of this preliminary study. Similarly, evaluating periapical healing potential necessitates in vivo studies to assess tissue responses over time. Consequently, we focused on core properties—sealing ability, antimicrobial efficacy, flow rate, and solubility—that can be quantitatively measured and directly influence the success of root canal treatments. Future research should include these critical factors to provide a more comprehensive understanding of the performance and long-term implications of root canal sealers.

### Key findings

In this study, Sealer B emerged as the top performer with the highest Clinical Utility Index of 95%, indicating superior overall performance across all four core properties. This finding strengthens existing literature, emphasizing the importance of balancing these properties to achieve optimal clinical outcomes. Sealer B demonstrated excellent antimicrobial efficacy, minimal solubility, optimal flow rate, and exceptional sealing ability, making it a highly recommended choice for endodontic practitioners.

Sealer C, with a Clinical Utility Index of 80%, showed strong performance, particularly in antimicrobial efficacy and flow rate, but was slightly less effective in sealing ability compared to Sealer B. This suggests that while Sealer C is still a viable option, improvements are needed in specific areas.

Sealers D and E, with lower Clinical Utility Indices of 30% and 35%, exhibited significant weaknesses in multiple properties. These results highlight the ongoing need for innovation in sealer formulations to address deficiencies and enhance clinical utility.

## Conclusion

The Clinical Utility Index developed in this study offers a robust and balanced framework for evaluating and ranking root canal sealers based on essential properties. This index facilitates better clinical decision-making by providing a comprehensive assessment of sealers' performance in sealing ability, antimicrobial effect, flow rate, and solubility. Sealer B's high performance underscores the importance of considering multiple properties in sealer selection. Future research should focus on refining this index, incorporating additional properties, and validating its applicability in diverse clinical scenarios. By continuously improving the criteria for sealer evaluation, we can enhance the quality and success of endodontic therapies.

## Data Availability

Data is provided within the manuscript.
